# Maize miRNAs Might Regulate Human Genes Involved in Prostate Cancer: An In Silico Approach

**DOI:** 10.3390/biotech14040095

**Published:** 2025-12-03

**Authors:** Ximena Vázquez-Cadena, Oscar Alejandro Faz-Cortez, Benito Pereyra-Alférez, César Ignacio Hernández-Vásquez, Luis Jesús Galán-Wong, Myriam Elías-Santos, Jorge Hugo Garcia-Garcia

**Affiliations:** Facultad de Ciencias Biológicas, Instituto de Biotecnología, Universidad Autónoma de Nuevo León, San Nicolás de los Garza 66455, Mexico; ximena.vazquezcdn@uanl.edu.mx (X.V.-C.); oscar.fazcr@uanl.edu.mx (O.A.F.-C.); benito.pereyraal@uanl.edu.mx (B.P.-A.); cesar.hernandezvsqz@uanl.edu.mx (C.I.H.-V.); luis.galanwn@uanl.edu.mx (L.J.G.-W.); myriam.eliassn@uanl.edu.mx (M.E.-S.)

**Keywords:** cross-kingdom regulation, microRNA, maize, prostate cancer, bioinformatics analysis

## Abstract

MicroRNAs (miRNAs) are small single-stranded non-coding RNA molecules that regulate gene expression at the post-transcriptional level. Recent studies have demonstrated that plant miRNAs can survive through dietary intake and act as signaling molecules in intercellular communication, proving a cross-kingdom interaction. The aim of the present study was to use computational approaches to identify interactions between *Zea mays* (maize) miRNAs and human coding mRNAs potentially involved in different biological processes. We identified 961 unique genes potentially regulated by maize miRNAs. Furthermore, functional enrichment analysis via GO and KEGG was carried out focusing primarily on the pathway related to prostate cancer where 13 genes were potentially regulated by 15 maize miRNAs. Our findings not only provide an important insight into the potential effects that maize-derived miRNAs could have on the human body, but also highlight the importance of considering these molecules for further research and potential therapeutic applications against major diseases such as cancer.

## 1. Introduction

MicroRNAs (miRNAs) are small noncoding RNA gene products about 19–22 nucleotides long derived from hairpin precursors that have been conserved throughout evolution [1] and are found in diverse organisms including animals and plants [2]. These molecules negatively regulate gene expression post-transcriptionally by pairing with complementary sequences in the CDS, 3′ UTR, and 5′ UTR regions of mRNAs, and through the RNA-induced silencing complex (RISC), which mediates mRNA cleavage or translation inhibition [3,4,5]. More than 60% of the proteins coded by the human genome are said to be regulated by miRNA [6].

The seed region, defined as nucleotides 2–7 at the 5′ end of the miRNA, plays a key role in binding to complementary sequences in target mRNAs. In animals, this region is typically fully complementary to the target site, whereas the remainder of the miRNA often exhibits only partial complementarity [7,8].

Numerous biological functions in humans, including behavior, stress reactions, hematopoietic cell differentiation, apoptosis, and organ development depend on miRNA-mediated gene control [9,10]. Cancer, heart disease, and metabolic disorders have all been connected to the dysregulation of miRNAs and the related protein machinery [11]. Beyond endogenous regulation, recent research has shown the existence of ‘cross-kingdom interactions,’ in which miRNAs from one species can influence gene expression in another [12]. Of particular interest are exogenous miRNAs derived from plants, which may be taken up by the epithelial cells of the gastrointestinal tract, repackaged into microvesicles, released into the bloodstream, and subsequently distributed to various mammalian organs even after the plant was cooked [13,14,15]. These miRNAs may influence the gene expression machinery inside the host cell [5,13,16].

In recent years, increasing attention has been given to plant miRNAs and their potential interactions with human mRNAs, shedding light on possible coevolutionary mechanisms between plant and animal [12,17]. A robust analysis showed that MIR168a, a plant-derived miRNA, is highly enriched in the sera of healthy Chinese individuals, and functional studies, both in vitro and in vivo, have shown that rice MIR168a can bind to the mRNA of the low-density lipoprotein receptor adapter protein 1 (LDLRAP1) in human cells and mice. This interaction appears to suppress LDLRAP1 expression in the liver, thereby reducing the clearance of LDL from mouse plasma [13].

Different studies have highlighted the effect of plant miRNAs on mammalian gene expression. For example, Hou et al. [18] showed that MIR156a—abundant in green vegetables—is detectable in human serum and can directly target the junctional adhesion molecule-A (JAM-A), which is upregulated in atherosclerotic lesions. Similarly, soybean gma-miR159a has been reported to suppress colon cancer cell growth in vitro and in vivo by targeting TCF7 [19], and the same miRNA was later shown to inhibit breast cancer cell proliferation in vitro [20].

Recently, two comprehensive reviews strengthened the concept of cross-kingdom gene regulation by dietary miRNAs. Yang and Feng [21] summarized accumulating experimental evidence demonstrating the absorption of exogenous plant miRNAs and their post-transcriptional activity in mammals, supporting their role as bioactive dietary components. Likewise, Shi et al. [16] synthesized bioinformatic evidence across multiple plant species, highlighting the potential impact of plant–human miRNA–mRNA interactions on human health.

Further investigation is required to gain a deeper understanding of this important cross-kingdom regulatory phenomenon, as the studies mentioned above highlight the potential impact that exogenous miRNAs acquired through dietary intake can have, as well as their promising therapeutic use against major diseases such as various types of cancer.

Maize is widely recognized for its nutritional and therapeutic potential. It contains a variety of bioactive compounds that have been linked to positive effects such as antioxidant, anti-inflammatory, anti-cancer, and cardioprotective effects, reflecting the complexity of maize as a food source [22]. Given that maize is one of the most consumed staple foods worldwide, we consider that understanding its molecular interactions within human biological systems is of great nutritional and biomedical relevance [23]. Therefore, in addition to its well-documented phytochemical components, exploring maize-derived miRNAs offers a complementary molecular perspective that may help explain some of the beneficial health effects traditionally attributed to maize consumption.

In the present study, we selected the *Zea mays* plant (maize) to predict potential human mRNA targets regulated by mature maize miRNAs, aiming to explore their possible functional implications. Our analysis revealed a wide range of predicted target genes involved in key cellular processes, including pathways related to prostate cancer (PCa). Among the enriched pathways, PCa was selected for deeper exploration due to its major clinical relevance and high global incidence and mortality rates in men [24]. Additionally, PCa has been previously linked to diet-dependent regulatory mechanisms, making it a biologically plausible candidate for exploring whether plant-derived miRNAs could modulate disease-related molecular networks [25]. Therefore, further analysis of these genes was carried out to gain insights into the potential relationship between maize consumption and the regulation of genes associated with tumor development in PCa, as well as to improve our understanding of the possible therapeutic role of miRNAs in the fight against cancer.

While previous studies have primarily examined cross-kingdom regulation in a limited number of plant species or individual miRNAs, to our knowledge no work has systematically evaluated the full repertoire of maize-derived mature miRNAs and their potential regulatory interactions with the human transcriptome. In this study, we integrate genome-wide target prediction with functional and pathway analyses, and further examine the presence of the identified maize miRNAs across other plant species as well as homology with the seed region of human miRNAs. This multi-layered approach enables us to prioritize biologically relevant interactions, with a particular emphasis on prostate cancer pathways, thereby expanding the current understanding of potential plant–human regulatory networks. Based on our results, we can hypothesize that specific *Z. mays* miRNAs possess sequence complementarity to human mRNA transcripts encoding key prostate cancer regulatory genes, thereby suggesting a potential regulatory mechanism.

## 2. Materials and Methods

### 2.1. Collection of miRNA Sequences

All mature miRNA sequences of *Z. mays* used in this study were obtained from the registered database (v22.1) https://www.mirbase.org (accessed on 13 January 2025). To enhance the quality of our analysis, we applied filtering criteria based on previous research [5,13]. Specifically, all selected mature miRNAs had to meet the following criteria: (1) more than 1400 reads reported in bioinformatics analyses, (2) a sequence length between 19 and 25 nucleotides, and (3) alignment in the same direction (5′ to 3′). After applying these filters, we refined our dataset from 325 available miRNAs to 137.

### 2.2. Prediction of Maize miRNA Targets in Human mRNA

To predict potential human mRNA targets of maize miRNAs, we first conducted an alignment between the 137 filtered sequences and the human (taxid:9606) RefSeq RNA database using the BLASTn (v2.9.0) sequence similarity search tool on the NCBI website http://www.ncbi.nlm.nih.gov (accessed on 16 January 2025). Default parameters of the “blastn-short” task were used, with an E-value threshold of 10 and low complexity filtering disabled (DUST = off). The search was restricted to nucleotides 2 to 13 of each miRNA, as previously reported [5]. Only alignments showing 100% sequence identity and >99% query coverage were retained. These thresholds were established based on the positive control, the interaction between *Oryza sativa* (rice) MIR168a and the human LDLRAP1 transcript, which displayed these same parameters and has been experimentally validated in previous studies [13].

Once the potential mRNA targets were identified using BLASTn (v2.9.0), their hybridization patterns with miRNAs were analyzed using RNAhybrid on the BiBiServ web server (build dated 18 September 2017) [26]. The following criteria were applied to select interactions for further analysis: (1) perfect matching in the seed region (nucleotides 2 to 7 of the mature miRNA), (2) a minimum free energy (MFE) below −25 kcal/mol, and (3) no G-U bonds in the seed region [5,27,28]. MIR168a targeting the LDLRAP1 transcript was used as a control for target prediction in these analyses [5,13]. We first obtained a list of human mRNAs potentially hybridized by maize miRNAs, which included non-coding transcripts and multiple mRNA isoforms corresponding to the same gene. For downstream analyses, we filtered this list using custom Python scripts (v13.3.6) based on Biopython (v1.86) [29], retaining only unique protein-coding genes by removing transcript isoforms from the same gene and excluding non-coding mRNAs.

### 2.3. GO and KEGG Pathway Enrichment Analysis

Once we obtained a list of unique genes without transcript isoforms from the same gene and excluding non-coding mRNAs, we predicted the biological effect of potential hybridization between maize miRNAs and human mRNAs. We conduced Gene Ontology (GO) and Kyoto Encyclopedia of Genes and Genomes (KEGG) pathway analyses using the online tool Enrichr https://maayanlab.cloud/Enrichr/# (accessed on 20 February 2025) [30,31]. We selected the ten most significant categories from each enrichment analysis, all with an adjusted *p*-value below 0.05 (FDR < 0.05).

### 2.4. Analysis of Genes Involved in Prostate Cancer

Prostate cancer-associated genes were retrieved from the KEGG prostate cancer pathway (hsa05215) identified by Enrichr. To visualize the interactions between maize miRNAs and genes encoded by hybridized mRNAs, Cytoscape software (v3.10.3) [32] was employed. Further analyses were performed using the 13 genes involved in PCa identified in the previous step. We used the Search Tool for the Retrieval of Interacting Genes/Proteins (STRING) database (v12.0) [33] to obtain a protein–protein interaction (PPI) network applying a high-confidence minimum required interaction score (0.700). This database integrates the BRENDA Tissue Ontology (BTO), which we used to perform a tissue-specific expression enrichment analysis to identify the expression levels of these genes. Tissues with FDR < 0.05 were considered significant.

To identify identical seed sequences between mature human miRNAs from the registered database (v22.1) https://www.mirbase.org (accessed on 23 February 2025) and maize miRNAs that potentially hybridize with mRNAs coding for the genes involved in PCa, as revealed by our previous results, we used custom Python scripts (v13.3.6) based on the Biopython library (v1.86) [29]. The human miRNAs with identical seed regions were analyzed using RNAhybrid [26] to predict hybridization patterns with the mRNAs of the 13 PCa-related genes targeted by the maize miRNAs. The following criteria were applied: no G-U bonds in the seed region, perfect seed region matching, and no restriction on the MFE. To determine whether the maize miRNAs predicted to hybridize with mRNAs encoding prostate-cancer-related genes are also found in other plant species, we retrieved the mature miRNA sequence dataset (mature.fa) from miRBase (release 22.1) https://www.mirbase.org (accessed on 2 November 2025). Identical matches (100% sequence identity across the mature sequence) were identified using custom Python scripts (v13.3.6) implemented with the Biopython library (v1.86) [29].

## 3. Results

### 3.1. Potential Hybridization of Human mRNAs by Maize miRNAs

**Figure 1:**
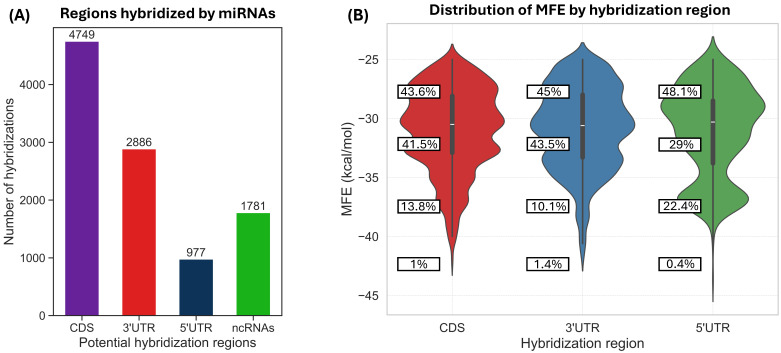
(**A**) Number of predicted hybridizations in each mRNA region (CDS, 3′ UTR, 5′ UTR and ncRNAs) among the 10,393 miRNA–mRNA interactions. (**B**) Distribution of MFE values for the interactions in the CDS, 3′ UTR, and 5′ UTR regions. Note that 22.4% of interactions in the 5′ UTR region exhibit MFE values between −35 and −40 kcal/mol, indicating strong potential binding.

We obtained 137 mature maize miRNA sequences following the filtering criteria previously described. According to our results using RNAhybrid, 129 of them showed at least one predicted interaction with a human mRNA, while 8 did not display any interaction under the criteria described in the Section 2. The total number of predicted hybridizations between human mRNAs and the 129 maize miRNAs was 10,393 (Table S1), of which 4749 occurred in coding sequences (CDS), 2886 in the 3′ UTR, 977 in the 5′ UTR, and 1781 in non-coding RNAs (Figure 1A).

All hybridizations showed a minimum free energy (MFE) value below −25 kcal/mol, as we discarded targets with higher MFE values, following previous recommendations [5]. The lower the MFE, the stronger the binding, and the more likely the mRNA target is to be a genuine target of the miRNA [34]. The lowest predicted MFE was −43.6 kcal/mol, corresponding to the hybridization of the miRNA zma-miR528a-3p with the mRNA encoding the *EXOC3L4* gene (Table S1). Most hybridizations fell within the −25 to −30 kcal/mol range (Figure 1B). However, it is worth noting that 22.4% of the interactions within the 5′ UTR exhibited binding energies in the −35 to −40 kcal/mol range, indicating strong potential hybridization.

The total of 10,393 predicted hybridizations was filtered to discard non-coding RNAs and gene variants. As a result, we obtained 961 unique genes with the potential to be regulated by maize miRNAs. Most genes are potentially targeted by more than one miRNA due to high sequence similarity or perfect sequence match among some miRNAs. Notably, some miRNAs are predicted to regulate a large number of genes, such as zma-miR529-5p (114 targets), zma-miR164a-5p and its homologs (77), and zma-miR528a-3p and zma-miR528b-3p (62) (Table S2).

Our predictions indicate that the miRNA osa-miR168a-5p from rice, used as a positive control [5], targets the *LDLRAP1* gene. This interaction has been confirmed by in vivo and in vitro analyses and was the first evidence of cross-kingdom miRNA transfer of this kind [13]. These findings support the methodology employed in our work. In fact, we found that the maize miRNAs zma-miR168a-5p and zma-miR168b-5p share an identical sequence with osa-miR168a-5p from rice. Based on the previously mentioned reports and our results, it is highly likely that these maize miRNAs also affect the *LDLRAP1* gene in vivo or in vitro.

### 3.2. Functional Enrichment Analysis of Genes Potentially Regulated by Maize miRNAs

**Figure 2:**
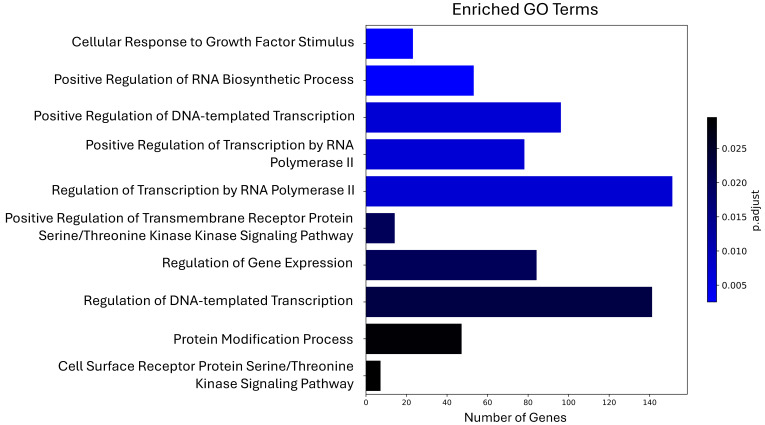
Top 10 most significant Gene Ontology terms enriched among human genes potentially regulated by maize miRNAs.

To identify the biological roles of the genes potentially regulated by maize miRNAs in the human organism, we performed Gene Ontology (GO) (Table S3) and Kyoto Encyclopedia of Genes and Genomes (KEGG) (Table S4) pathway analyses. Using the 961 unique genes as input, we reported the 10 most significant terms or pathways from each analysis. The GO terms corresponding to biological processes revealed several categories with a high number of enriched genes, such as regulation of transcription by RNA polymerase II (151 genes), regulation of DNA-templated transcription (141 genes), and positive regulation of DNA-templated transcription (96 genes) (Figure 2).

**Figure 3:**
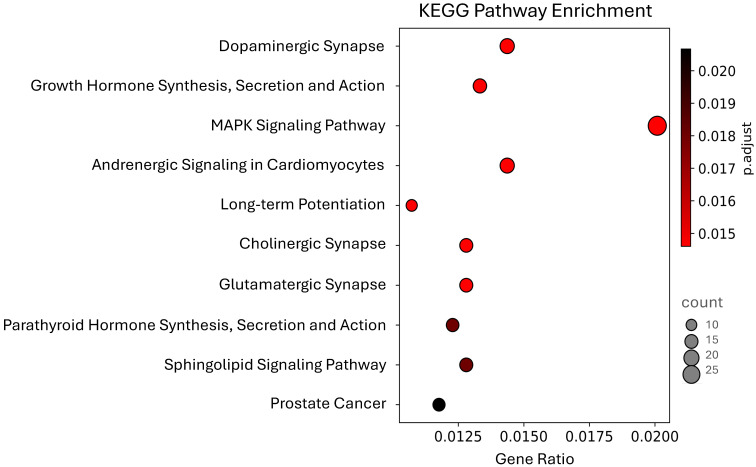
The top 10 most significant KEGG Pathways enriched among human genes potentially regulated by maize miRNAs.

The KEGG analysis revealed three pathways related to synaptic processes: cholinergic synapse, glutamatergic synapse, and dopaminergic synapse (Figure 3). That last one has previously been reported as enriched with genes potentially regulated by wheat miRNAs [5]. Our results showed that 32 genes are involved in more than one of the ten pathways we identified (Table S5). The *PRKCA* gene is present in all of them except for the PCa pathway. Additionally, the genes *PLCB4*, *GNAQ*, and *ATF4* are involved in eight pathways, while *CACNA1C*, *GNAI2*, and *AKT3* appear in seven of the ten reported pathways.

The MAPK signaling pathway was the most enriched, with 29 genes; however, we also identified 13 genes enriched in the PCa pathway. Given the significant impact of this disease, which is associated with high mortality rates in the male population [24], we decided to further analyze these genes to explore a potential relation between maize consumption and PCa, as well as improve our understanding of the possible therapeutic role of miRNAs in the fight against cancer.

Previous studies have reported maize-derived compounds with anticancer activity against PCa, such as maysin, anthocyanins, and flavonoids [35,36,37]. These findings suggest that maize contains bioactive molecules capable of modulating PCa. However, there is a lack of studies evaluating PCa in relation to maize miRNA interactions. For this reason, together with the high clinical relevance of PCa and its previously reported association with diet-dependent regulatory mechanisms [25], we selected this pathway for deeper analysis.

### 3.3. Maize miRNAs Potentially Regulate Human Genes Involved in Prostate Cancer

**Figure 4:**
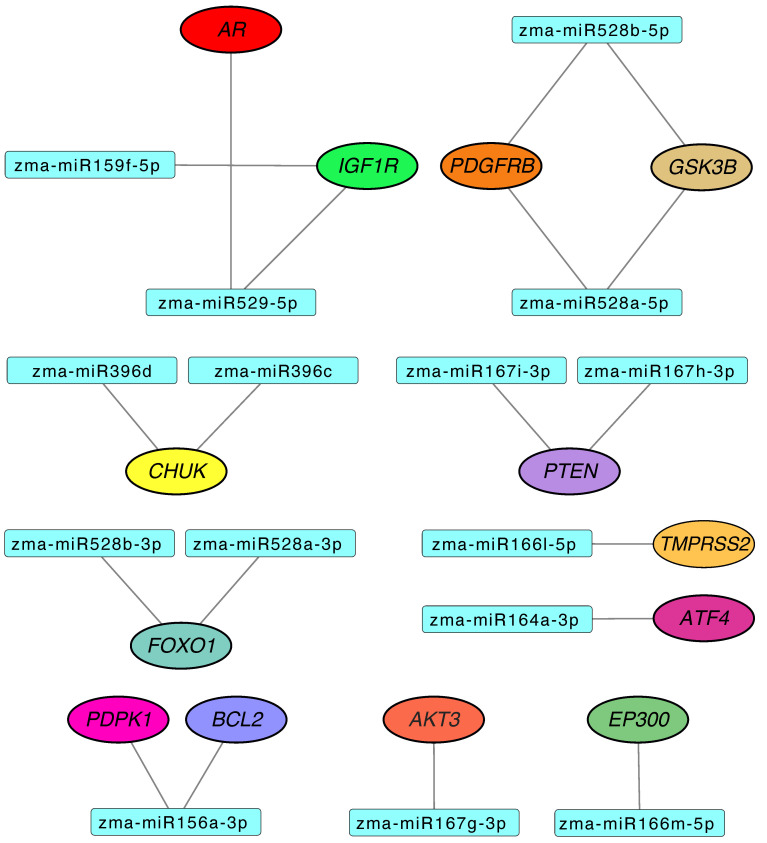
Predicted interactions between maize miRNAs and the 13 human genes involved in PCa and potentially regulated by these miRNAs.

KEGG analysis of the 961 genes predicted as targets of maize miRNAs revealed 13 involved in the PCa pathway, regulated by 15 distinct miRNAs (Figure 4). The mRNA–miRNA binding sites were located in the 3′ UTR for eight genes, in the 5′ UTR for four genes, and in the CDS region for one gene. Seven of these interactions showed MFE lower than −30 kcal/mol, corresponding to the *BCL2*, *IGF1R*, *PDGFRB*, *GSK3B*, *TMPRSS2*, *FOXO1*, and *CHUK* genes (Table 1), suggesting they may represent the most promising candidates for future functional validation.

Some genes appeared to be regulated by two different maize miRNAs. This dual binding is due to high or identical sequence identity between the miRNAs, with hybridization occurring at the same mRNA position. However, the *IGF1R* gene is targeted at two different positions by two different miRNAs. Additionally, miRNAs such as zma-miR529-5p, zma-miR528b-5p, zma-miR528a-5p, and zma-miR156a-3p are capable of hybridizing with two different genes within the pathway (Figure 4).

To identify the expression levels of these 13 genes, we performed a tissue-specific expression enrichment analysis. The results support their involvement in PCa, as we observed significant enrichment in tissues such as prostate gland cancer cells, adenocarcinoma cell lines, VCaP cells and DU-145 cells, cells-based models of human prostate cancer [38,39]. This analysis also revealed significant expressions of five genes in cervical carcinoma cells (Table 2). Although the KEGG analysis did not show significance in pathways related to this cancer, the activity of these five genes might still play a crucial role in cervical cancer biology.

**Figure 5:**
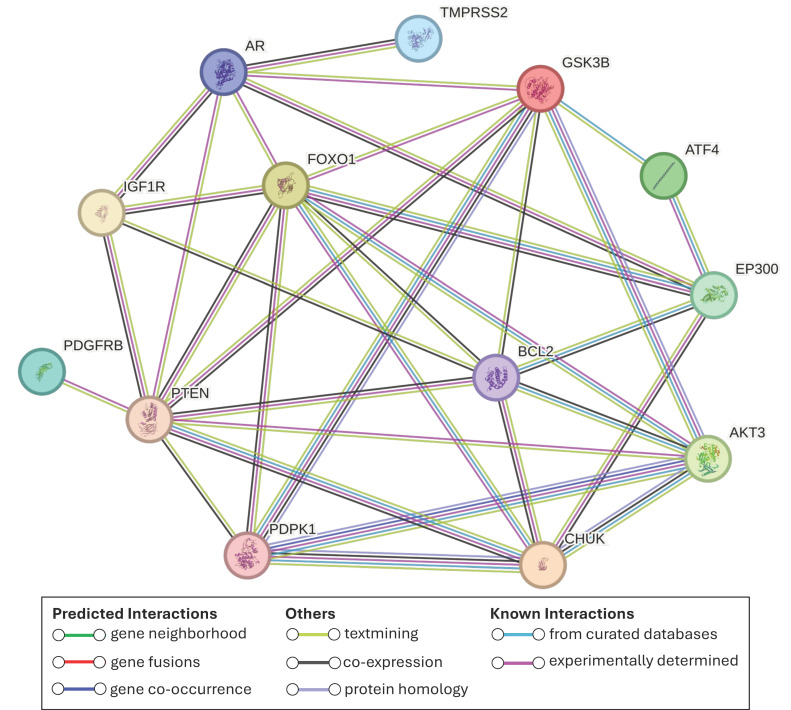
PPI network among the 13 proteins involved in PCa and potentially regulated by maize miRNAs. Note that proteins such as PTEN and FOXO1 exhibit 9 interactions, while GSK3B and BCL2 show 7 each, highlighting their crucial roles within the pathway.

To determine potential interactions among the 13 proteins, we performed a protein–protein interaction (PPI) network analysis. The network revealed that all proteins are involved in at least one interaction with another protein from the group (Figure 5). Some proteins exhibited a substantial number of interactions, such as PTEN and FOXO1, each with 9 connections, and GSK3B and BCL2, each with 7, highlighting their crucial roles in the pathway.

To assess functional homology between maize miRNAs potentially involved in the regulation of PCa-related genes and human miRNAs, we searched for human miRNAs with identical seed regions. We identified 14 human miRNAs that share an identical seed sequence with maize miRNAs predicted to target genes associated with PCa. To evaluate the potential hybridization between these 14 human miRNAs and the corresponding PCa-related genes (also targeted by maize miRNAs), as well as to estimate their MFE, we used RNAhybrid. We found predicted hybridizations for 5 mRNAs corresponding to 5 out of the 13 PCa-related genes (Table 3).

All hybridizations between human miRNAs and gene transcripts occurred in the same regions as their maize miRNA homologs: the 5′ UTR for *PTEN* and the 3′ UTR for *BCL2*, *PDPK1*, *GSK3B*, and *PDGFRB*. Most predicted hybridizations had MFE values above −25 kcal/mol; only two were below this threshold. The *GSK3B* gene showed potential binding with the hsa-miR-5008-3p miRNA. Similarly, the *BCL2* gene appeared to be targeted by the human hsa-miR-1304-3p, with a particularly strong hybridization (−38.5 kcal/mol). Although several of the predicted interactions had relatively high MFE values, the possibility of mimetic effects between exogenous maize miRNAs and endogenous human miRNAs should not be overlooked.

Finally, we evaluated whether the maize miRNAs predicted to hybridize with mRNAs encoding prostate cancer-related genes are also found in other plant species. Our results show that 11 out of the 15 maize miRNA sequences are present in other plants (Table 4), which is particularly interesting because some of these species—such as *Sorghum bicolor*, *Saccharum* sp., and *Oryza sativa*—are consumed by humans.

## 4. Discussion

Nutrition is no longer understood just as the intake of macro and micronutrients, but also as a source of bioactive molecules that can modulate gene expression. Among them, plant-derived miRNAs have gained attention for their ability to cross the gastrointestinal barrier and potentially influence gene regulation in animals even after cooking [13,15]. These findings suggest that dietary miRNAs may represent an additional layer of dietary influence on human health.

In this study, we used bioinformatics analysis to explore potential cross-kingdom interactions between maize miRNAs and human mRNAs. We started our analysis with 137 selected maize miRNAs using BLASTn to search for complementary sequences within the human RefSeq transcriptome. This allowed the prediction of multiple target genes and their variants, offering a more systematic view of how plant miRNAs might impact human gene regulation [40]. These results were then analyzed using RNAhybrid, focusing only on interactions with a MFE below −25 kcal/mol and perfect seed region complementarity between the miRNA and mRNA. This resulted in 10,393 predicted miRNA–mRNA interactions involving 129 miRNAs out of the initial 137 sequences (Table S1). These interactions are distributed across CDS, 3′ UTR, 5′ UTR regions, and non-coding RNAs, illustrating the wide range of possible regulatory targets. After excluding gene variants and non-coding RNAs, we identified 961 unique genes potentially regulated by maize miRNAs (Table S2).

We found that three of the maize miRNAs predicted to target human mRNAs share identical sequences with wheat miRNAs (tae-miR167a, tae-miR164, and tae-miR159a), which were analyzed in a previous study [5] and showed similar gene targets, although our analysis predicted a broader range.

Of particular interest, we also identified that two maize miRNAs, zma-miR168a-5p and zma-miR168b-5p, share an identical mature sequence with osa-miR168a-5p from rice. This perfect sequence identity is not only relevant from a regulatory perspective, but also provides evolutionary evidence of strong conservation within Poaceae (the grass family). Such conservation implies that these miRNAs likely originated from a common ancestral *locus* and have retained selective pressure across monocots, preserving both sequence and function [41].

Notably, osa-miR168a-5p has been experimentally shown to regulate the human *LDLRAP1* gene in vivo and in vitro [13]. The fact that maize orthologs exhibit an identical mature sequence suggests that the regulatory potential observed in rice may reflect a broader, evolutionarily conserved mechanism among grasses. From a phylogenetic standpoint, this supports the idea that plant miRNAs can maintain cross-kingdom regulatory capabilities when their key functional motifs particularly the seed region are preserved across species, it is highly likely that these maize miRNAs could similarly affect *LDLRAP1* expression in vivo or in vitro, as maize miRNAs have specifically been detected crossing the gastrointestinal tract, entering the bloodstream, and distributing across tissues in pigs, targeting endogenous genes even after the maize was cooked [42]. In fact, it has been previously reported that higher maize intake is associated with a lower prevalence of risk of hypertension [43], hypertriglyceridaemia [44], and lower concentrations of total and low-density lipoprotein cholesterol [44,45]. Our analysis, supported by the findings of Zhang et al. [13], suggests that maize miRNAs may represent one of the contributing bioactive components in maize potentially mediating these effects.

Furthermore, functional enrichment analysis via GO and KEGG was carried out on the 961 unique genes potentially regulated by maize miRNAs. We identified pathways related to PCa, transcription factor activity, and synaptic functions such as cholinergic, glutamatergic, and dopaminergic synapses, which were previously predicted to be regulated by wheat miRNAs [5]. These results suggest that plant miRNAs may influence a broad array of cellular processes, including those implicated in cancer and neuronal signaling [46,47].

PCa has been previously linked to diet-dependent regulatory mechanisms [25], which makes it a biologically plausible candidate for examining whether plant-derived miRNAs could modulate disease-related molecular networks. For this reason, along with the significant impact of PCa on human health [24], we focused our analysis and discussion on this disease.

At the genetic level, several alterations have been reported as key in PCa, such as *TMPRSS2-ETS* fusions, amplification of the *MYC* oncogene, mutations or amplification of *AR*, and mutations or deletions of *PTEN* and *TP53* [48]. Our enrichment analysis identified 13 genes associated with the PCa pathway, potentially regulated by 15 different miRNAs (Figure 4).

One of these 13 genes is *TMPRSS2*, which is of particular interest due to its frequent fusion with *ETS* family genes, especially *ERG*, forming one of the most common and aggressive alterations in PCa [49]. This fusion, absent in normal tissue, is androgen-regulated and associated with poor prognosis [50]. If plant miRNAs repress *TMPRSS2* expression, they could potentially reduce fusion formation and tumor progression without affecting healthy tissues. The TMPRSS2 protein appears functionally integrated with the AR protein (Figure 5), suggesting that its modulation could have broader downstream effects within the androgen signaling axis.

Complementing the role of *TMPRSS2* in androgen regulation, additional predicted targets participate in signaling axes such as PI3K/Akt. For instance, *PTEN*, a tumor suppressor predicted as a target of two maize miRNAs (zma-miR167h-3p and zma-miR167i-3p). Its loss promotes tumorigenesis and is commonly suppressed by human miRNAs such as miR-19b and the miR-106b~25 cluster [51,52]. We also identified *PDGFRB* as a target of two maize miRNAs (zma-miR528a-5p and zma-miR528b-5p), which simultaneously target *GSK3B*. According to our analysis, these miRNAs share 100% seed region identity with five human miRNAs (Table 3), suggesting a potential mimicry effect.

Another relevant gene within this network is *BCL2*, whose anti-apoptotic activity and documented overexpression in PCa have been linked to resistance to cell death and disease advancement [53]. Our predictions also identified this gene as a target of zma-miR156a-3p. Its regulation by miRNAs has been reported, such as in the case of miR-204-5p, which has been shown to promote apoptosis in PCa cells [53,54,55]. In our analysis, we identified hsa-miR-1304-3p, a human miRNA with an important low MFE (−38.5 kcal/mol) (Table 3), sharing the same seed region as zma-miR156a-3p, suggesting a potential mimicry effect. Although hsa-miR-1304-3p has not been reported to target *BCL2* directly, Zhao et al. [56] demonstrated its role in cancer by the repression of *GATA2*, a gene that plays a complex role in the development of this disease [57]. We propose that the predicted interaction between zma-miR156a-3p and *BCL2* could represent a beneficial regulatory mechanism capable of fighting cancer progression, especially considering the established role of *BCL2* in tumor cell survival.

Beyond apoptosis-related mechanisms, androgen signaling remains a central axis in PCa biology. In this context, we identified the androgen receptor (AR) as a predicted target. Despite androgen deprivation, AR remains active in castration-resistant prostate cancer (CRPC), sustaining tumor growth [58,59]. Coactivators such as CBP and EP300 interestingly, the latter also predicted as a target in our study, enhance AR-mediated transcription and are associated with advanced disease [60]. In addition, GSK3B, a kinase that modulates AR signaling and was among our predicted targets, has been shown to promote apoptosis when inhibited in PCa cells [61].

Expanding on this, our PPI analysis revealed functional connections between AR, IGF1R, and FOXO1, pointing to a tightly regulated signaling network. It has been reported that IGF1R activation induces FOXO1 phosphorylation, causing its exclusion from the nucleus and thereby suppressing its ability to inhibit AR activity [62]. Supporting this, IGF1R has been shown to be upregulated during androgen-independent progression of PCa [63,64]. Similarly, our PPI network also showed interactions between FOXO1 and GSK3B. In line with this, previous studies have demonstrated that GSK3B can directly phosphorylate FOXO1, leading to its inactivation [65].

Furthermore, PTEN and FOXO1 displayed the highest number of connections, with nine interactions each, followed by GSK3B and BCL2, which had seven, highlighting their potential central roles in the regulation of PCa-related pathways.

Additional predicted targets involved in the PI3K/AKT pathway include PDPK1 and AKT, which play key roles in cell proliferation, apoptosis, and survival [66,67,68,69]. Broader regulators such as CHUK and ATF4, associated with inflammation and stress response, were also identified [70,71]. *CHUK* encodes IKKα, a central kinase of the IKK complex that activates NF-κB signaling by promoting IκBα degradation, thereby influencing processes such as immune regulation, cell survival, and tumorigenesis [72]. Meanwhile, ATF4, a transcription factor in the unfolded protein response (UPR) [73], has been shown to be essential for PCa growth, with increased expression in tumors and a critical role in maintaining tumor survival through its downstream effector FAM129A [74]. Also, these same authors showed that in vivo therapeutic silencing of the *ATF4-FAM129A* axis significantly inhibits tumor progression, highlighting its potential as a therapeutic target.

Although some studies have associated maize consumption with certain types of cancer [75,76], other research has identified anticancer properties in specific peptides and bioactive compounds isolated from maize [35,77,78,79]. These findings reflect the complexity of maize as a food source and highlight the need to explore additional molecular components such as miRNAs that may also contribute to its biological effects. In this study, we identified several miRNAs that could have beneficial effects against cancer, such as one that potentially downregulates the *BCL2* gene, which encodes an anti-apoptotic protein and is frequently overexpressed in various cancer types. Conversely, some miRNAs may act as potential onco-miRNAs by targeting tumor suppressor genes such as *PTEN* [80].

Our results not only suggests that maize-derived miRNAs represent a previously unexplored layer of gene regulation with potential implications for cancer biology but also indicate a putative relationship between maize miRNAs and human gene regulation overall. The in silico analysis provides valuable initial insight into the potential physiological effects of maize consumption on human physiology, particularly in diseases such as cancer. These findings highlight the significance of computational approaches as a foundation for exploring cross-kingdom gene regulation between plant-derived miRNAs and humans. We also showed that several important maize miRNAs are conserved in other plant species such as *S. bicolor*, *B. distachyon* and *O. sativa*, all of which share close evolutionary relationships within Poaceae. This includes miR528, which has an identical mature sequence in multiple grasses, suggesting conserved regulatory functions among monocots [81]. One additional species fell outside Poaceae, indicating that conservation is not restricted to grasses (Table 4).

Importantly, we identified that several of the maize miRNAs predicted to target PCa-related genes share seed regions that are identical to human miRNAs that interact with the same transcripts. This sequence-level similarity may help explain why certain plant miRNAs appear capable of binding to human genes such as *PTEN*, *GSK3B*, *PDGFRB*, *PDPK1*, and *BCL2* (Table 3). Instead of reflecting random complementarity, these patterns could be influenced by evolutionary constraints that maintain conserved miRNA seed architectures across species. While experimental validation is required, this potential “functional mimicry” supports the plausibility of cross-kingdom interactions and suggests that some maize miRNAs may resemble endogenous human miRNAs in the way they engage with specific regulatory sites. Nonetheless, experimental validation remains essential to confirm our predictions and to further elucidate the role of dietary miRNAs in human health and disease.

Our analysis revealed that several maize miRNAs potentially target key regulators of prostate-cancer signaling, including *PTEN*, *IGF1R*, *AR*, *FOXO1*, *GSK3B*, and *BCL2*. These genes play central roles in androgen signaling, PI3K/AKT regulation, apoptosis, and cell-cycle control, suggesting that the predicted cross-kingdom interactions converge onto well-established oncogenic pathways. While computational, these findings identify a coherent biological theme and generate mechanistic hypotheses—such as the possibility that plant-derived miRNAs could modulate androgen-receptor activity or AKT-mediated survival signaling—that warrant experimental exploration. Rather than presenting isolated matches, our work highlights conserved and pathway-integrated regulatory modules, thereby advancing the field beyond previous reports that examined only individual miRNAs or lacked pathway-level interpretation.

## 5. Conclusions

This study provides a computational framework to explore potential cross-kingdom regulatory interactions between maize-derived miRNAs and human mRNAs. Our in silico predictions identified possible hybridizations involving 961 human genes, many of which participate in key biological processes such as synaptic signaling, apoptosis, and major cancer-related pathways, including PI3K/AKT and androgen signaling. Among these, 13 genes were associated with the prostate cancer pathway and showed potential regulation by 15 maize miRNAs. These findings provide important exploratory insight into the potential effects that maize-derived miRNAs could have on the human body and might serve as a starting point for identifying biologically meaningful candidate interactions. They also highlight the possible therapeutic relevance of miRNAs in cancer research. However, they remain predictive and require rigorous experimental validation to demonstrate functional transfer and biological activity.

## Figures and Tables

**Table 1:** Genes involved in PCa and potentially regulated by maize miRNAs.

Gene	Hybridization Region	MFE (kcal/mol)
*AR*	5′ UTR	−25.7
*BCL2*	3′ UTR	−32.9
*IGF1R*	5′ UTR	−30.9
	5′ UTR	−28.4
*PDGFRB*	3′ UTR	−33.4
*GSK3B*	3′ UTR	−38.2
*PDPK1*	3′ UTR	−28.3
*TMPRSS2*	3′ UTR	−37.3
*ATF4*	5′ UTR	−26.8
*EP300*	CDS	−25.7
*AKT3*	3′ UTR	−29.3
*FOXO1*	3′ UTR	−30.2
*PTEN*	5′ UTR	−29.4
*CHUK*	3′ UTR	−30.8

**Table 2:** Tissues significantly enriched by the expression of genes involved in PCa.

Term Description	Gene Count	False Discovery Rate	Genes
Prostate gland cancer cell	3	6.51 × 10^−5^	*PTEN*, *AR*, *BCL2*
Cervical carcinoma cell	5	0.0017	*EP300*, *GSK3B*, *CHUK*, *FOXO1*, *IGF1R*
VCaP cell	2	0.0032	*AR*, *TMPRSS2*
DU-145 cell	2	0.0051	*AR*, *BCL2*
Adenocarcinoma cell line	3	0.0225	*AR*, *BCL2*, *TMPRSS2*
Prostate cancer cell line	3	0.0225	*AR*, *BCL2*, *TMPRSS2*
Lymphocytic leukemia cell	4	0.0389	*EP300*, *GSK3B*, *ATF4*, *PDPK1*

**Table 3:** Predicted hybridizations between human miRNAs (sharing identical seed regions with maize miRNAs) and PCa-related genes.

Genes	Target ID	Human miRNA(s)	Hybridization Region	MFE (kcal/mol) for Human miRNAs
*PTEN*	NM_000314.8	hsa-miR-96-3p	5′ UTR	−22
		hsa-miR-2681-3p	5′ UTR	−20.1
*BCL2*	XM_054318967.1	hsa-miR-1304-3p	3′ UTR	−38.5
*PDPK1*	XM_047434201.1	hsa-miR-1304-3p	3′ UTR	−20.7
*GSK3B*	NM_002093.4	hsa-miR-5008-3p	3′ UTR	−26.5
		hsa-miR-6737-3p	3′ UTR	−19.9
		hsa-miR-6889-3p	3′ UTR	−17.4
		hsa-miR-7157-3p	3′ UTR	−23.4
		hsa-miR-6529-3p	3′ UTR	−19.6
*PDGFRB*	NM_002609.4	hsa-miR-5008-3p	3′ UTR	−23
		hsa-miR-6737-3p	3′ UTR	−15.5
		hsa-miR-6889-3p	3′ UTR	−16
		hsa-miR-7157-3p	3′ UTR	−19.3
		hsa-miR-6529-3p	3′ UTR	−18.7

**Table 4:** Maize miRNAs predicted to hybridize with mRNAs encoding prostate cancer-related genes found in other plant species.

Maize miRNA	Also Found in
zma-miR159f-5p (GAGCUCCUCUCAUUCCAAUGA)	Not detected in other plant species
zma-miR529-5p (AGAAGAGAGAGAGUACAGCCU)	*Brachypodium distachyon*
zma-miR528b-5p zma-miR528a-5p (UGGAAGGGGCAUGCAGAGGAG)	*Sorghum bicolor*; *Brachypodium distachyon*; *Saccharum* sp.; *Aegilops tauschii*; *Vriesea carinata*; *Oryza sativa*
zma-miR396c zma-miR396d (UUCCACAGGCUUUCUUGAACUG)	*Sorghum bicolor*
zma-miR167h-3p zma-miR167i-3p (GAUCAUGUUGCAGCUUCAC)	*Brachypodium distachyon*; *Oryza sativa*
zma-miR528a-3p zma-miR528b-3p (CCUGUGCCUGCCUCUUCCAUU)	*Aegilops tauschii*; *Brachypodium distachyon*
zma-miR166l-5p (GAAUGGAGGCUGGUCCAAGA)	Not detected in other plant species
zma-miR164a-3p (CACGUGUUCUCCUUCUCCAUC)	Not detected in other plant species
zma-miR156a-3p (GCUCACUUCUCUCUCUGUCAGU)	Not detected in other plant species
zma-miR167g-3p (GGUCAUGCUGUAGUUUCAUC)	*Oryza sativa*
zma-miR166m-5p (GGAAUGUUGGCUGGCUCGAGG)	*Medicago truncatula*; *Oryza sativa*

Presence based on exact mature-sequence identity comparison against miRBase v22.1.

## Data Availability

The original contributions presented in this study are included in the article/Supplementary Materials. Further inquiries can be directed to the corresponding author.

## Supplementary Materials

The following supporting information can be downloaded at: https://www.mdpi.com/article/10.3390/biotech14040095/s1, Table S1: The total number of predicted hybridizations between human mRNAs and the maize miRNAs; Table S2: Interactions between maize miRNAs and human mRNAs; Table S3: GO analysis; Table S4: KEGG analysis; Table S5: Genes involved in more than one pathway.

## Abbreviations

The following abbreviations are used in this manuscript:

miRNA	MicroRNA
CDS	Coding sequence
RISC	RNA-induced silencing complex
LDL	Low-density lipoprotein
PCa	Prostate cancer
GO	Gene ontology
MFE	Minimum free energy
KEGG	Kyoto Encyclopedia of Genes and Genomes
UTR	Untranslated region
PPI	Protein–protein interaction
FDR	False discovery rate
