# Supplementary material for: Maize miRNAs Might Regulate Human Genes Involved in Prostate Cancer: An In Silico Approach
Source: BioTech (Basel). 2025 Dec 3;14(4):95. doi: 10.3390/biotech14040095 (PMC12730536; doi:10.3390/biotech14040095)
Supplement: Supplementary file 1 [file biotech-14-00095-s001.zip › Table S2.pdf]

Table S2. Interactions between maize miRNAs and human mRNAs.

miRNA (s)	Number of target genes	Target genes
zma-miR156d-5p<br>zma-miR156f-5p<br>zma-miR156g-5p<br>zma-miR156b-5p<br>zma miR156c<br>zma-miR156e-5p<br>zma-miR156a-5p<br>zma-miR156h-5p<br>zma-miR156i-5p<br>zma-miR156l-5p<br>zma-miR156k-5p	22	ADGRD1, ALG14, BTBD7, CCDC15, CCDC88C, EML4, ERN1, EXT2, F11R, FAM13A, GMPR, KIF5A, LMCD1, LVRN, MAPK11, SFMBT2, TGOLN2, TMEM201, TRAPPC9, TTBK1, TTN, ZBTB40
zma-miR164a-5p<br>zma-miR164d-5p<br>zma-miR164b-5p<br>zma-miR164c-5p<br>zma-miR164g-5p<br>zma-miR164e-5p<br>zma-miR164f-5p<br>zma-miR164h-5p	77	ABCB9, ACBD4, AKR1C8, APC2, APOBEC3A, APOBEC3A_B, APOBEC3B, ARHGAP33, B3GNT7, BEAN1, BIRC6, BTBD19, CACNG5, COMMD5, CSRP1, DLG4, FLYWCH1, FRY, FRYL, FSIP1, FSTL4, FTO, GABBR1, GLI3, GNAI2, GREM1, GRM2, IFT172, KCNA2, KCNH6, KIF1A, LOC105371253, LOC124900193, LOC124902561, LOC124906647, LRRC55, MLEC, MLLT6, MNT, MTCL1, MYOT, NCAPH, NDUFA10, PAQR9, PBXIP1, PCYT2, PDE1C, PIP5KL1, POU2F2, PPP3CA, PPRC1, RAP1GAP2, RASSF6, RFPL4B, RFT1, SCN4B, SETD4, B4GALNT2, SLC25A1, SLC6A7, SLC9A9, SMARCC2, SNAPC4, SNED1, SYNE1, TAC4, TAF8, TGFB3, TLR2, TNFRSF13C, TRIM41, TSPYL4, TTLL4, UBE2I, VPS72, ZNF689, ZNF701
zma-miR167a-5p<br>zma-miR167b-5p<br>zma-miR167d-5p<br>zma-miR167c-5p	42	BCL7B, CACNA2D4, CACNB3, CBX7, CRADD, EPPK1, FLRT1, FOXN1, GOLGA8F, GOLGA8S, IGSF5, KCNQ3, LMO1, LOC124903407, LOC124907502, LOC128966784, LTBP4, MAML2, MAPT, MBD3, MCM7, MDM1, NCBP3, NOX5, PI4KB, PIP5KL1, PLET1, PRICKLE2, RPS6KA1, SEC14L1, SLC35E2B, SLC9B2, SPESP1-NOX5, SPHK1, TCEANC2, THBS3, TM2D3, TNFRSF10D, WASHC2A, XPA, ZNF469, ZNF530
zma-miR166a-3p<br>zma-miR166h-3p<br>zma-miR166e<br>zma-miR166i-3p<br>zma-miR166f<br>zma-miR166g-3p	3	DNMBP, FBXL6, LOC400499

zma-miR166b-3p<br>zma-miR166c-3p<br>zma-miR166d-3p<br>zma-miR166k-3p<br>zma-miR166n-3p<br>zma-miR166j-3p<br>zma-miR166l-3p<br>zma-miR166m-3p		
zma-miR171b-3p<br>zma-miR171f-3p	6	DNAJC5G, PGAP6, RPAP1, TMEM151A, ZIC2, NAT16
zma-miR172a<br>zma-miR172d-3p<br>zma-miR172b-3p<br>zma-miR172c-3p	11	CCDC7, CHST5, EPB41, FOXB2, LSM8, NSMAF, PLEC, PRPF18, TREM2, TTN, ZNF438
zma-miR159a-3p<br>zma-miR159b-3p<br>zma-miR159f-3p<br>zma-miR159j-3p<br>zma-miR159k-3p	17	CCSER1, CNOT9, EDA, GPR135, GPR4, LOC124903407, LOC128966784, MAN1A2, OR2F1, PLCB4, PTPRC, RFC3, RSPH4A, SNTB1, TEX13C, THEMIS, TMEM232
zma-miR319a-3p<br>zma-miR319c-3p<br>zma-miR319b-3p<br>zma-miR319d-3p	19	ACLY, CCDC134, CEP104, COL16A1, DLG4, FAT4, FBLIM1, KLC2, KMT5A, LRRK2, MUC16, PHF1, RAB5B, SCART1, SLC1A4, SPATA31F3, ST8SIA2, TASP1, TIGD7
zma-miR167e-5p<br>zma-miR167f-5p<br>zma-miR167g-5p<br>zma-miR167h-5p<br>zma-miR167i-5p<br>zma-miR167j-5p	43	BCL7B, CACNA2D4, CACNB3, CBX7, CRADD, EPPK1, FLRT1, FOXN1, GOLGA8F, GOLGA8S, IGSF5, KCNQ3, LMO1, LOC124903407, LOC124907502, LOC128966784, LTBP4, MAML2, MAPT, MBD3, MCM7, MDM1, MYRF, NCBP3, NOX5, PI4KB, PIP5KL1, PLET1, PRICKLE2, RPS6KA1, SEC14L1, SLC35E2B, SLC9B2, SPESP1-NOX5, SPHK1, TCEANC2, THBS3, TM2D3, TNFRSF10D, WASHC2A, XPA, ZNF469, ZNF530
zma-miR168a-5p<br>zma-miR168b-5p<br>osa-miR168a-5p (C)	12	ADCY9, PARG, CPED1, HIVEP3, LDLRAP1, NOVA2, SLC38A1, SPEN, TNFAIP8, WHRN, ZNF66, SPOCK3
zma-miR169j-5p	21	CACNA1D, CD84, CHST10, COL25A1, EEIG2, EXOC3L1, F11R, GALNT17, KRTAP21-2, MAP3K20, MBTPS1, PDK1, RHCE, RHD, RNF169, SETBP1, SNRK, SPTBN2, THEMIS, TUBB1, ZNF749
zma-miR396c<br>zma-miR396d	22	ABCA9, AFG1L, CHUK, DIAPH3, DRC7, EPHA6, FGGY, HAS2, IL17RD, IL24, JMJD6, LOC124901892, MAPK14, MARCHF6,

		MBNL3, PCNX1, PIP4K2A, PWWP2A, TTBK1, VAT1L, ZFP62, ZNHIT3
zma-miR528a-5p zma-miR528b-5p	35	ACVR2B, AGO2, ARFRP1, ARID5A, C14orf39, CDON, CTSV, EFL1, ELMO1, FAM174B, FANCC, FIBCD1, GSK3B, HIVEP3, IFITM10, LZTS2, NOP9, OBSCN, PDGFRB, PMEPA1, PPFIBP2, PTPN18, RABEP2, REL, RHBDF2, SLC28A1, SLC6A11, SMAD1, TMEM150B, TMEM184B, TPCN1, TRIO, USP48, ZIC3, ZKSCAN2
zma-miR529-5p	114	ADAMTS17, PARP11, ALOX15, AMOT, ANKRD44, AR, ARB2A, ATP11A, BMP2K, BMP3, BRWD3, C20orf203, C22orf42, C8orf33, CASTOR1, CIART, CREBZF, CUX1, DPF3, DPY19L1, DUSP16, DUSP7, DZIP1L, EGR1, ELAPOR2, ELF4, ENSA, EPB41, FAM20B, FAP, FBXL7, FLRT2, FMOD, FOSL2, FSIP2, FTO, FYN, GABPA, GCFC2, GLIS3, HMGA2, IGF1R, IL1RAP, KATNAL1, KCNA5, KIF26B, LCP1, LDOC1, LINGO2, LMBRD1, LOC105374341, LOC124900420, LOC124901780, LOC124906014, LOC124906582, LRP5, LRRC18, LYN, MAGI2, MAP3K15, MAST4, MORC2, MSANTD2, MSH2, MUC16, MUCL3, MZF1, NAALADL2, NAV2, NDUFA2, NFAT5, NFIA, NHSL2, NR1H4, NR2F1, NSD1, OCA2, OPCML, OR51G2, OR56A4, P3H2, PLCH2, PPM1A, PPP1R21, PRRT2, RFC3, RFPL1, SCD, SCFD2, SCP2, SETBP1, SETD1B, SMIM14, SNAPC3, SND1, SPACA9, SPRY2, STX3, SWAP70, SYNE3, ERMAP, THBS1, TMEM156, TRIM4, TRIM67, USB1, VWA5B1, XYLT1, ZFHX3, ZHX2, ZMYM3, ZNF292, ZNF446, AOPEP
zma-miR156a-3p	35	AVL9, BCL2, BRWD3, CERCAM, CYGB, DOCK11, EPB41, FAM163A, FMN1, FTO, GABRQ, GNAZ, GPR101, HIVEP2, HPCAL1, LMNTD1, LOC124909461, MAPKAPK2, MSI2, PDPK1, PRRG3, PTPRD, RAP1A, RBBP8NL, ROR1, SLC6A11, SMAD4, SNX19, SS18L2, TACC2, TCL1A, TMX4, ZNF490, ZNF621, ZNF705G
zma-miR156b-3p	5	HIPK2, MBD5, RIMKLA, USP54, ZHX2
zma-miR156h-3p	42	ARPC5L, BCAR1, BSPRY, CCDC120, CCDC14, CHFR, CLIC4, CNNM4, DAG1, DOCK10, DOT1L, ENDOD1, ENOX1, FANCI, FARS2, FBXW2, GDF2, GPR146, DNAJC27, IDS, IMMP2L, JMJD1C, MAS1, MTG2, MTR, PAX5, PRKCH, RALGAPA2, RALY, RBM45, SBF1, SERPINB9, SLC17A5, SP140, STAMBP, TAF8, TUSC3, WNT5A, XKR6, ZNF284, ZNF490, ZNF683
zma-miR156e-3p	49	ABCB4, AGBL5, AP1G2, ASPHD1, ATP5IF1, BSDC1, CCDC180, CDH2, CES3, CHAT, CNNM3, CSN1S1, CTNNA1, DHX16, DMP1, HAS3, HIF3A, HIRIP3, IMPG2, JMJD1C, KCNH2, MPO, NAB1,

		NFKBID, NLRC3, OCSTAMP, PAX5, PDE4D, PPOX, PPP1R2C, PPP3CB, PTPRF, RABL6, RILP, RLIM, SCFD2, SF3B1, SLC2A13, SLIT1, SYNE2, TCOF1, TJP2, TMBIM1, TMEM161B, TRAPPC9, TRMO, UBAP1L, UNC93A, ZNF385D
zma-miR156d-3p<br>zma-miR156f-3p<br>zma-miR156g-3p	27	ADAMTSL1, ALK, ATAD2B, CD300C, CEP128, CFAP97D2, COX11, CTNND1, IGSF10, IQGAP2, LAMB3, LOC124907782, LPP, MAGI2, MTARC1, NME6, NOPCHAP1, NSD3, NUBPL, OR51G2, ROR1, SIPA1L2, STRBP, STT3B, STX7, SULF1, THRB
zma-miR156i-3p	15	ATP7A, CHSY1, CMIP, DNAH9, HEBP2, LOC107987292, MINDY3, NCKAP1L, OR56A3, RNF40, RPAP1, SERTAD2, SIRT3, TUSC3, XKR6
zma-miR164a-3p	5	ATF4, KLHL42, SLC28A3, SOX9, TCF3
zma-miR164b-3p	26	AJAP1, ATG7, CAMKK2, CEP57L1, COBL, GRIN2B, GRIP2, HELB, IMPG2, IP6K2, KCNJ9, KPNA1, LENEP, LRP1, NAALADL2, PLEKHA5, PRR23E, PRSS33, RPS4X, SPRY4, TRIML2, WDR25, WDR27, YEATS4, ZNF74, ZNF775
zma-miR164d-3p	14	CABS1, CD180, DBH, LOC124900388, LOC728392, MARF1, MPPE1, MYOC, PITPNM2, RTN2, SH3BP2, SMIM24, SUMF1, WDPCP
zma-miR827-3p	4	ALMS1, MMD2, TMEM232, ZYX
zma-miR164c-3p<br>zma-miR164h-3p	15	AKAP13, ANKRD50, ATP13A3, BMPER, BORCS5, CDIN1, EXO5, HDAC4, MAP4K1, MS4A2, NALF2, PLA2R1, RAP1GAP, RSRP1, TUT7
zma-miR166a-5p<br>zma-miR166g-5p<br>zma-miR166b-5p<br>zma-miR166c-5p<br>zma-miR166d-5p	7	CERCAM, DCC, IKZF2, MCC, OSMR, RAG2, WRAP73
zma-miR166h-5p	1	HSF2BP
zma-miR167a-3p	1	CDKAL1
zma-miR167b-3p	11	AREG, BRD10, DMXL2, FUT9, GLDC, INSYN1, MRPL19, TBC1D12, TMPO, TUBGCP6, PTK7
zma-miR167c-3p	13	ASXL3, BHMT2, CYP1B1, DSE, LIG3, NYAP1, OPRL1, PDE4D, PPP2R5C, SASH1, SMARCA2, SMARCA4, UQCRFS1
zma-miR167d-3p	37	AFF2, BAG3, CEACAM16, CNDP2, EHF, PIEZO1, FN1, FYN, GPRC5C, IFT57, ILF3, KCNJ9, KCNN3, KYAT1, KYAT1-SPOUT1, LOC124900173, MAD1L1, MOB3B, MTNR1B, NHSL1, NR1I2, PILRA, PPDPF, PRKDC, RALGAPB, RIN2, SCN2A, ST6GAL2, TESPA1, TLE2, TLE3, TTC28, TXNRD2, UBR4, USP11, ZDHHC7, ATP9B

zma-miR172d-5p<br>zma-miR172b-5p	18	APPL2, ARSL, ATP6V1A, BTBD16, COL3A1, DENND1B, DMTF1, GGCX, HMGCS1, HSPE1-MOB4, MOB4, OR56A1, OR5AS1, SERPINA4, SOX13, TBCD, TFRC, ZSCAN25
zma-miR159a-5p	5	GBP5, LOC124904958, SMYD3, TAAR5, TNS1
zma-miR159b-5p<br>zma-miR159j-5p<br>zma-miR159k-5p	9	CFAP299, DMRT2, ELL, ETS1, ITK, NOTCH2, WWTR1, ZNF169, ZNF451
zma-miR166j-5p	14	BCL11A, CEP128, FNDC3B, H2AC20, MAP3K13, NPAS2, NR6A1, PALM2AKAP2, PLXDC2, SCUBE1, SLC39A8, SLCO4A1, TASP1, TBC1D2
zma-miR166k-5p	4	ATP8B4, CCSER1, CT83, CUX2
zma-miR167e-3p	9	ACAD11, ANKDD1B, C1QTNF3, JMJD1C, NAALADL2, NSFL1C, RAE1, SERTAD4, USP19
zma-miR167g-3p	7	AKT3, C1orf50, JAK2, MAP4K4, NPAS3, NWD1, TM4SF4
zma-miR167h-3p<br>zma-miR167i-3p	8	CECR2, CWC22, FADS2, GCSH, KDM4C, PTEN, SLC15A5, ZMAT1
zma-miR168a-3p	4	CECR2, FADS2, INTS6, ZNF25
zma-miR168b-3p	4	ANKRD49, FSD2, IMMP2L, SOX30
zma-miR171d-5p<br>zma-miR171e-5p	6	BMP2, CAMSAP1, EHMT1, MPZL1, PSG1, PSG3
zma-miR171f-5p	20	BECN2, CACNA1C, CRYBG1, FTO, GPR18, MUC17, NAALADL2, OR52A1, PELI2, PSKH1, RELCH, RGPD5, RGPD6, SEZ6, SGK1, SHISA6, SRD5A1, WHRN, ZNF462, ZNF74
zma-miR172c-5p	18	C8G, CNTNAP2, ESRP1, GPATCH8, ITGA1, LAMA5, LDLRAD4, LOC124903827, LOC128966581, LRTM2, MTSS2, PDSS2, PSEN2, SCN8A, SIGLEC5, TMEM26, TTN, ZFYVE27
zma-miR319a-5p<br>zma-miR319c-5p	27	SLC29A1, CPSF2, DCLRE1C, DNAAF19, DNAH11, ESR1, GFAP, GON4L, IKZF5, LOC124903713, LOC124904942, LYPD6, MKRN2, NDUFA5, NEK10, NEO1, OR5M1, PANK2, PDE3A, PDE4C, PTMS, SGSM1, SSX2IP, TNPO2, TTC7B, UBASH3A, ZNF462
zma-miR319b-5p<br>zma-miR319d-5p	6	AMZ1, CPSF2, PPL, SPMIP7, TRPA1, WDR27
zma-miR156k-3p	12	BHMT2, CENPJ, CPAP, CRNN, EPC1, FECH, MTSS1, SMARCA4, SNRPB, SPEG, UPF3A, ZMYM3
zma-miR156l-3p	14	ATP7A, CHSY1, CMIP, DNAH9, HEBP2, LOC107987292, MINDY3, NCKAP1L, OR56A3, RNF40, RPAP1, SERTAD2, SIRT3, XKR6
zma-miR159f-5p	19	CYB5R4, DCHS1, FAM135B, GCSAM, GLYATL2, HMGXB3, IGF1R, INTS6, LOC124905687, MTUS2, MUC4, NLRP3,

		PIP5K1C, RFLNB, RIPPLY2-CYB5R4, SERPIND1, SLC25A19, SPTBN2, TENT4A
zma-miR164e-3p	4	F2R, GDF11, KMT2D, PPP1R26
zma-miR164f-3p	1	ASXL1
zma-miR164g-3p	6	ACKR3, ERICH1, GPR146, PGS1, PNPLA7, SFMBT2
zma-miR166l-5p	23	ABCC11, ABLIM3, ALDH7A1, ASAP1, C20orf96, CFAP46, DCBLD2, DISP3, EYS, FRMD5, ICMT, IMMP2L, MSI2, NUDT3, PDE1C, PPRC1, REL, ROR1, SIPA1L1, SLC30A8, TMPRSS2, TSPAN12, ZNF469
zma-miR166m-5p	16	ACSM2A, ACSM2B, APOBEC3A_B, C16orf82, COLEC12, DRC8, ECPAS, EP300, ETV6, HIVEP1, MRGPRE, NPAS2, PCLO, RALGAPA2, ZNF551, ZNF892
zma-miR166n-5p	4	ATP8B4, CCSER1, CT83, CUX2
zma-miR167j-3p	8	APOL3, CHEK1, ESCO2, FHOD3, GAN, GNAQ, TBCK, TREML4
zma-miR171i-5p	6	AMOTL1, MAGED4, MAGED4B, MAGEL2, MBLAC2, TACC2
zma-miR444a<br>zma-miR444b	7	MBD4, NCOA1, SEMA4A, SLC6A15, SLC6A17, VPS26B, WDTC1
zma-miR528a-3p<br>zma-miR528b-3p	62	ADAMTSL1, ADCY3, AHNAK2, AHRR, ANKRD13B, APCDD1L, ASPG, ATG7, ATXN7L3, CCDC120, CCDC13, CCDC85C, CERK, CFLAR, CHST15, CPA3, DCHS1, EXOC3L4, FILIP1L, FOXO1, FYCO1, GAN, GREB1L, GRIA4, HDAC6, HES2, HOXB3, KLF2, KPNA5, LGALS9, LMOD1, LRRFIP1, MED26, GALNS, NRSN1, OTOG, PHETA1, PLEKHG2, POP1, PPIL2, PRKCA, PTP4A3, PTPA, RD3, RNPEPL1, RUNX2, SBF1, SFRP1, SLC22A7, SLC66A1, SLC6A11, SMTN, STAB1, SV2C, SYT3, TEX13A, THSD4, TNFSF12, TNFSF12-TNFSF13, VSX1, ZCCHC18, ZNF623
zma-miR529-3p	9	BTBD7, CRMP1, ENOX1, GATAD2B, IQCB1, LRRCC1, PCGF5, SETBP1, ZNF212
zma-miR827-5p	3	CNTN4, GTPBP1, PTPN12
